# Expression of truncated Int6/eIF3e in mammary alveolar epithelium leads to persistent hyperplasia and tumorigenesis

**DOI:** 10.1186/bcr1742

**Published:** 2007-07-12

**Authors:** David L Mack, Corinne A Boulanger, Robert Callahan, Gilbert H Smith

**Affiliations:** 1 Mammary Biology and Tumorigenesis Laboratory, National Cancer Institute, National Institutes of Health; Bethesda, Maryland, 20892, USA

## Abstract

**Introduction:**

Int6 has been shown to be an interactive participant with the protein translation initiation complex eIF3, the COP9 signalosome and the regulatory lid of the 26S proteasome. Insertion of mouse mammary tumor virus into the Int6 locus creates a C-terminally truncated form of the protein. Expression of the truncated form of Int6 (Int6sh) in stably transfected human and mouse mammary epithelial cell lines leads to cellular transformation. In addition, decreased expression of Int6/eIF3e is observed in approximately one third of all human breast carcinomas.

**Methods:**

To validate that Int6sh has transforming activity *in vivo*, a transgenic mouse model was designed using the whey acidic protein (Wap) promoter to target expression of truncated Int6 to differentiating alveolar epithelial cells in the mammary gland. Microarray analyses were performed on normal, premalignant and malignant WapInt6sh expressing tissues.

**Results:**

Mammary tumors developed in 42% of WapInt6sh heterozygous parous females at an average age of 18 months. In WapInt6sh mice, the contralateral mammary glands from both tumorous and non-tumorous tissues contained widespread focal alveolar hyperplasia. Only 4% of WapInt6sh non-breeding females developed tumors by 2 years of age. The Wap promoter is active only during estrus in the mammary tissue of cycling non-pregnant mice. Microarray analyses of mammary tissues demonstrated that Int6sh expression in the alveolar tissue altered the mammary transcriptome in a specific manner that was detectable even in the first pregnancy. This Int6sh-specific transcriptome pattern subsequently persisted in both the Int6sh-expressing alveolar hyperplasia and mammary tumors. These observations are consistent with the conclusion that WapInt6sh-expressing alveolar cells survive involution following the cessation of lactation, and subsequently give rise to the mammary tumors that arise in aging multiparous females.

**Conclusion:**

These observations provide direct *in vivo *evidence that mammary-specific expression of the Int6sh truncation leads to persistence of alveolar hyperplasia with the accompanying increased predisposition to mammary tumorigenesis.

## Introduction

Int6/eIF3e (p48) was originally isolated from a mammary hyperplastic outgrowth cell line derived from a preneoplastic hyperplastic alveolar nodule (HAN), its tumors and metastases, and two independently arising mammary tumors [1]. In each clone, the mouse mammary tumor virus (MMTV) integrated in an intron of one allele of Int6 in the reverse transcriptional orientation to that of Int6, generating a chimeric mRNA. Integration events were found in introns 5, 9 and 12 producing different C-terminal truncations of Int6. The most extreme truncation (in intron 5) produced an mRNA containing sequences encoding the N-terminal 137 amino acids of Int6 (out of 445 amino acids), novel sequences from intron 5 upstream of the integration site and reverse sequences from the MMTV 3' LTR upstream of the cryptic stop signal, Int6 short (Int6sh). The essentially random integration of retroviral DNA, and the fact that Int6 was mutated in virtually the same way in multiple independent tumors, suggests its potential role in malignant transformation. No mutations in the remaining Int6 allele were detected in these MMTV-induced tumors, and the expression levels of full-length Int6 transcripts appeared unchanged, suggesting that the truncation creates a dominantly acting mutation.

Int6 has been highly conserved throughout evolution. It encodes the p48 subunit of the eukaryotic translation initiation factor-3, eIF3 subunit e. This large protein complex is responsible for dissociating 80S ribosomes into subunits and promoting the binding of methionyl-tRNA and mRNA to the 40S ribosomal subunit during the initiation phase of protein synthesis [2]. In fission yeast, Int6/eIF3e co-purifies with the eIF3 complex but is not essential for protein translation, suggesting that this subunit plays a regulatory role [3,4]. Additional functions emerging for Int6/eIF3e include regulating protein turnover through its binding to the regulatory lid of the 26S proteasome [5] and the COP9 signalosome [6]. Yin6 (yeast ortholog of Int6) positively regulates the 26S proteasome, which functions to degrade polyubiquitinated proteins, by binding to and mediating the nuclear import and assembly of another proteasome regulatory subunit, Rpn5 [7]. The resulting degradation of polyubiquitinated proteins is believed to be essential for progression through the cell cycle. In addition to the proteasome, Int6 also associates with the COP9 signalosome, CSN. In the single-celled yeast, the CSN regulates the cell cycle checkpoint but in multi-cellular organisms, including plants, it also participates in multiple developmental pathways, which are all dependent on its control of ubiquitin-mediated protein degradation by the proteasome [8,9]. The COP9 signalosome has been shown to have several additional activities: de-ubiquitination, protein kinase and metalloprotease activities, each believed to be contributing to the regulation of ubiquitin-proteasome-mediated protein degradation [10].

Direct evidence for the oncogenic activity of Int6sh *in vitro *came from forced expression experiments showing that a truncated form of Int6 can transform cells in culture [11]. Two mammary epithelial cell lines, MCF10A (human) and HC-11 (mouse), expressing Int6sh from the elongation factor promoter (eEF1a), exhibited anchorage-independent growth in soft agar. Using slightly different criteria for cellular transformation, Mayeur and Hershey confirmed the *in vitro *transforming activity of the Int6 truncation by stably transforming mouse fibroblasts with a version of Int6 truncated after exon 4. They also showed that fibroblasts expressing their truncated form of Int6 were resistant to serum starvation-induced apoptosis [12]. In addition, transplantation of MCF10A-Int6sh cells into cleared fat pads of athymic mice led to the development of epithelial nodules in half the fat pads. Similarly, HC11-Int6sh cells produced lobular/alveolar structures at a rate of ~10% when injected into cleared fat pads of Balb/c mice. When HC11-Int6sh cells were transplanted into filled fat pads similar lobular/alveolar structures arose in 20% of the fat pads, suggesting that the HC11-Int6sh cells could overcome local growth regulatory control usually observed in a filled fat pad in a manner similar to pre-malignant epithelial cells [11]. Taken together, the *in vitro *and *in vivo *data strongly suggest an indirect role for Int6 in proliferation and cell cycle control.

To determine whether the *in vitro *transforming ability of Int6sh carries over to new *in vivo *tumor development, a transgenic mouse line was created with mammary-specific Int6sh expression. The original preneoplastic mammary lesions and tumors from which Int6sh was isolated harbored two to four additional MMTV insertions. It remains possible that one or more of these unidentified MMTV-induced mutations cooperated with the Int6 truncation to produce the observed hyperplastic alveolar nodules and tumors. To test this and model the role of the Int6 mutation in alveolar hyperplasia, we targeted Int6sh expression to the differentiating alveolar epithelium using the whey acidic protein (Wap) promoter, which is under tight hormonal regulation in the mouse mammary gland [13,14]. Wap expression occurs mainly during the secretory development of the mammary gland during late pregnancy and during lactation. Our WapInt6sh transgenic model demonstrates that ectopic expression of a truncated form of Int6 from the Wap promoter in the mammary epithelium results in persistent alveolar hyperplasia leading to mammary tumorigenesis.

## Materials and methods

### Generation and maintenance of WapInt6sh transgenic mice

**Figure 1:**
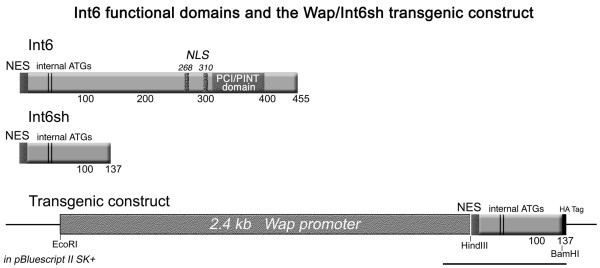
Int6 functional domains and wapint6sh transgenic construct. A schematic of the Int6 protein is shown: NES, nuclear export signal; internal ATGs denote alternative start sites located approximately 40 amino acids downstream of the predominant start site; NLS, bipartite nuclear localization sequences at amino acid numbers 268 and 310; PCI/PINT domain, proteasome, COP9 signalosome, initiation factor 3/proteasome subunits, Int-6, Nip-1 and TRIP-15 domain characteristic of proteins that make up the proteasome, signalosome and translation initiation complexes. The number of amino acids in each protein is shown below the bars. The extent of the Int6sh truncation is shown for comparison. The construct used to generate the WapInt6sh transgenic mice is shown at the bottom. A PCR-amplified *Hin*dIII/*Bam*HI fragment was cloned downstream of the 2.4 kb Wap promoter fragment in pBluescript SK+. HA Tag, hemagglutinin epitope tag. The line at the lower right designates the 602-bp junction fragment amplified by RT-PCR. The sizes of DNA fragments are not drawn to scale.

A 472-bp *Hin*dIII/*Bam*HI cDNA fragment encoding the truncated form of Int6 (amino acids 1–137), with an in frame hemagglutinin (HA) epitope tag at its C-terminus, was subcloned immediately downstream of the 2.4-kb whey acidic protein (Wap) promoter in pBluescript II SK+ (Stratagene, La Jolla, CA, USA) (Figure 1). The construct was linearized, gel-purified and microinjected into pronuclei of embryos from FVB/N mice. Microinjected embryos were transferred to the oviducts of pseudo-pregnant FVB/N females. Transgene-positive founder mice were identified by subjecting tail-snip genomic DNA to PCR using the following primers to amplify a 602-bp Wap promoter/Int6sh junction fragment (Primers: Wap promoter 2,281 forward, 5'-TGGCCAAGAAGGAAGTGTTGTAGCC-3'; HA Tag 2,883 reverse, 5'-GCGTAATCCGGTACGTCATATGGG-3'). Although two founders were originally identified, female offspring from only one of the 14 founders (J1 male) showed consistent Int6sh mRNA expression by RT-PCR in their mammary glands during late pregnancy and bred successfully. Therefore, all results reported herein are from one transgenic founder. This founder and its progeny were backcrossed with FVB/N mice from our colony that have been genetically isolated for more than 10 years. All mice were housed in Association for Assessment and Accreditation of Laboratory Animal Care-accredited facilities in accordance with the NIH Guide for the Care and Use of Laboratory Animals.

### Mammary gland histology

Whole mounts of thoracic and inguinal mammary glands were prepared by spreading the glands on a glass slide and fixing in Carnoy's fixative (10% glacial acetic acid, 30% chloroform and 60% absolute ethanol) for 4 h to overnight. Following fixation, the glands were stained with 0.2% carmine alum, washed with 70% ethanol for at least 30 min, followed by 95% ethanol for at least 1 h. Glands were defatted in xylenes for at least 30 min and mounted under coverslips using permount. For histological examination, whole mounts were embedded in paraffin, sectioned at 6.0 μm and stained with hematoxylin and eosin.

For the analysis of mammary glands from WapCreRosa26stopWapInt6sh mice, whole mounts were fixed in 4.0% paraformaldehyde for 1–2 h, permeabilized in 0.01% Nonidet P-40 in PBS overnight at 4°C and incubated with X-Gal substrate (1 mg/ml) overnight at 37°C. Stained glands were repeatedly rinsed in PBS, postfixed in Carnoy's fixative, dehydrated in 100% ethanol and cleared in xylenes. For histological examination, X-Gal-stained glands were embedded in paraffin, sectioned at 6.0 μm, and counterstained with nuclear fast red.

### RNA preparation and RT-PCR

Mammary-specific Int6sh expression was then determined by the RT-PCR amplification of a fragment spanning the Wap promoter/Int6sh junction. RNA was prepared from wild-type and Int6sh transgenic mammary glands using the Qiagen Lipid Tissue Kit according to the manufacturer's protocol (Qiagen, Valencia, CA, USA). DNaseI-treated total RNA (1 μg) was subjected to the SuperScript™ First-Strand Synthesis System for RT-PCR (Invitrogen, Carlsbad, CA, USA). Then, one tenth of that reaction was amplified by target-specific linear PCR (20–25 cycles) for GAPDH and Int6sh using Platinum Taq DNA Polymerase (Invitrogen). Int6sh PCR primers were the same as those employed for mouse genotyping. Control primers for the housekeeping gene, GAPDH were as follows: forward, 5'-ACCACAGTCCATGCCATCAC-3'; reverse, 5'-TCCACCACCCTGTTGCTGTA-3'. Band intensities on 1.2% agarose gels reflected the relative amount of each transcript present in the original sample.

To determine if Int6sh message was present in hyperplastic alveolar nodules, HANs were dissected away from the surrounding normal mammary gland and total RNA from the tumor, HANs and normal tissue were amplified separately. Equal amounts of total RNA were used in each first-strand synthesis reactions, followed by the target-specific PCR method outlined above.

### Microarray analysis

Additional file 1 summarizes the pooling scheme from wild-type and transgenic mammary glands and two of the three tumor types and shows the microarray hybridizations performed. Total RNA was prepared from each gland separately, quantitated and quality tested. Only then was the same amount of RNA (1 μg) combined to make an individual pool. From each pool 1 μg of total RNA was converted into labeled cRNA with nucleotides coupled to a fluorescent dye (either Cy3 or Cy5) using the Low RNA Input Linear Amplification Kit (Agilent Technologies, Palo Alto, CA, USA) following the manufacturer's protocol. The quality and quantity of the resulting labeled cRNA was assessed using a NanoDrop ND-1000 spectrophometer (NanoDrop Technologies, Wilmington, DE, USA) and an Agilent 2100 Bioanalyzer. Equal amounts of Cy3 and Cy5-labeled cRNA (750 ng) from two different samples were hybridized to mouse microarrays (Agilent Mouse Oligo Microarrays, G4121A) for 17 h at 60°C. The hybridized array was then washed and scanned using an Agilent G2565AA scanner. Data was extracted from the scanned image using Feature Extraction v. 7.1 or 7.5 (Agilent Technologies; Redwood City, CA, USA) and analyzed using GeneSpring v. 7.2 software (Agilent Technologies). Each hybridization was performed in duplicate in the form of Cy3/Cy5 dye flips (Additional file 1) and a standard deviation was calculated for each pairwise comparison (data not shown). This limited statistical power made it essential for us to validate the microarray data via quantitative RT-PCR.

### Validation of microarray data by qRT-PCR

Equal amounts of total RNA from wild-type and transgenic mammary glands (distinct from that prepared for the microarray hybridizations) were pooled as outlined in Additional file 1 and then treated with DNaseI. Each pooled sample was then quantitatively converted to single stranded cDNA using the High-Capacity cDNA Archive Kit (Applied Biosystems). The reaction from each pool was then quantitated using a NanoDrop spectrophotometer and 100 ng of the cDNA reaction products were added to 18 individual target-specific TaqMan Gene Expression Assays (TaqMan MGB probes, FAM dye-labeled according to the manufacturer's protocol) using GAPDH and ACTB as endogenous references and TaqMan Universal PCR Master Mix (Roche Molecular Systems, NJ, USA). All reactions were performed using a 96-well format on a Stratagene Mx3000P Quantitative PCR Instrument and analyzed using MxPro software v. 3.0. Reactions for the endogenous controls were performed in quadruplicate while the target-specific reactions were performed in duplicate. Standard curves over six orders of magnitude were performed to confirm that the amplification efficiencies of all target genes were similar to both endogenous controls. The comparative C_T _method for relative quantitation was employed to generate fold-change values for each of the 18 genes, normalized independently against GAPDH and ACTB. Statistical analyses were performed according to the manufacturers guidelines (Real-Time PCR Systems Chemistry Guide, Applied Biosystems).

## Results

### WapInt6sh transgenic founders transgene expression and tumorigenesis

Figure 1 shows a schematic of the functional domains of Int6 indicating those deleted in the truncated transgenic construct. A total of 14 WapInt6sh founder mice and their first generation offspring were screened by RT-PCR for mammary-specific Int6sh expression. Four founder females expressed Int6sh in their mammary glands, but all late pregnant female offspring failed to consistently express Int6sh. Five female offspring from one founder male (J1) consistently showed Int6sh expression in their mammary glands and successfully passed the transgene to their progeny. As expected, the whey acidic protein (Wap) promoter successfully targeted expression of Int6sh to mammary epithelial cells. Additional file 2 demonstrates that expression of the transgene was induced in pregnant females at approximately day 15 of gestation with sustained expression through parturition and early involution. The J1 founder male and his female offspring were backcrossed to wild-type FVB/N mice to generate the F1s that were then interbred to produce the mice analyzed in this study. The original J1 founder male developed a testicular tumor (where Wap expression has also been demonstrated [15]), and metastases in the liver and pancreas, all of which tested positive for Int6sh expression (data not shown).

### WapInt6sh multiparous females developed persistent hyperplastic alveolar nodules and mammary tumors consistent with malignant progression

**Figure 2:**
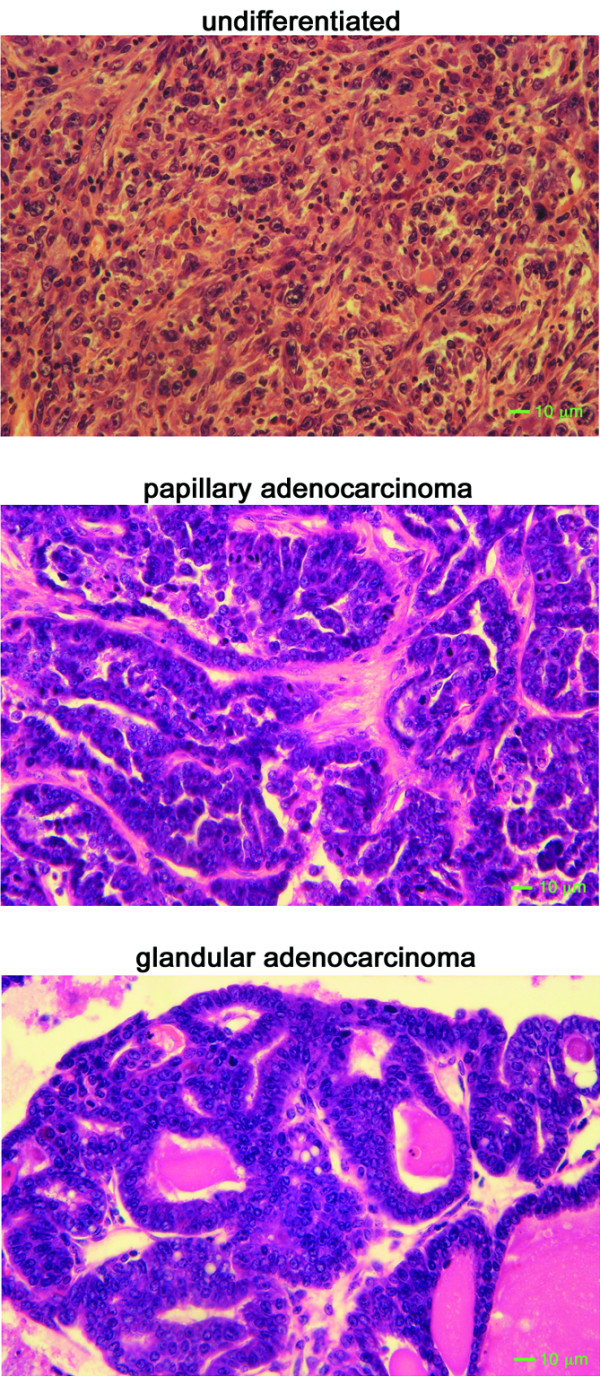
Representative histopathologies of three different types of mammary tumors arising in WapInt6sh multiparous females. The top panel shows an undifferentiated tumor; the middle panel shows the features of a papillary adenocarcinoma, including fibrovascular stalks covered in neoplastic epithelium; the bottom panel is more indicative of a glandular adenocarcinoma. Sections 5 μm thick were hematoxylin and eosin stained and photographed at 200 × magnification. A 10-μm bar is shown at the lower right of each panel.

Heterozygous transgenic Int6sh female FVB/N mice developed tumors at a frequency of 41.7% (10/24) around 18 months of age after giving birth to several (2–4) litters (Table 1). Despite a long tumor latency, these tumors grew quite rapidly once established. Prior to tumor formation, the multiparous Int6sh transgenic female mice were able to reproduce and to lactate normally. Histologically, three types of the tumors arose in the Int6sh multiparous females. One contained only undifferentiated epithelia, a second group showed characteristics of papillary adenocarcinoma while a third group appeared more glandular (Figure 2). The incidence of each tumor type was approximately equal (tumor incidence data are summarized in Additional file 3). In contrast to multiparous females, only one nulliparous Int6sh transgenic female mouse of the same age developed a tumor (1/26 = 3.8%), consistent with the decreased expression of the Wap promoter in nulliparous females. This tumor histologically resembled the papillary adenocarcinomas. In an earlier study from our lab, 22 multiparous female FVB mice (from the same FVB inbred subline used to construct the WapInt6sh model) were held for tumor incidence for more than 2 years with no spontaneous mammary tumors [16]. The Fisher's exact test p value reported for the tumor incidence comparison between the wild-type multiparous littermates and WapInt6sh multiparous females, p = 0.035, is probably conservative given the historical multiparous controls with a p value of 0.0006. Based on the low mammary tumor incidence reported in the bulk of the literature (summarized in [17]), the equally low spontaneous mammary tumor incidence in multiparous FVB female mice in our isolated colony [18], the identification of the MMTV-induced Int6 truncation in a pre-neoplastic mammary lesion and the ability of Int6sh to transform cells in culture, we believe these tumors were a direct result of expression of Int6sh.

**Table 1:** Mammary tumor incidence in WapInt6sh female transgenic mice compared with wild-type female FVB/N mice at 24 months of age.

Strain and genotype	No. of mice with tumors	Total mice (N)	Tumor incidence (%)	Fisher's exact test p value	Reference
FVB/N wild-type^a^	0	71	0	<0.00001^b^	[17]
FVB/N wild-type, multiparous^c^	0	22	0	0.0006^d^	[16]
FVB/N WapInt6sh virgins	1^e^	26^f^	3.8%	0.0016^g^	This work
FVB/N wild-type, multiparous^h^	0	8	0	0.035^i^	This work
FVB/N WapInt6sh, multiparous	10^j^	24^k^	41.7%	NA	This work

^a^Reported by the National Institute for Environmental Health Sciences. For this comparison, only histological malignant lesions were scored as tumors; adenomas and other benign lesions were not included.

^b^p < 0.00001 from the Fisher's exact test of previously published tumor incidence in FVB/N wild-type mice vs WapInt6sh multiparous females (row 1 vs row 5).

^c^Includes all frank tumors classified histopathologically as adenomas or adenocarcinomas.

^d^p = 0.0006 from the Fisher's exact test of previously published tumor incidence in FVB/N wild-type multiparous mice vs WapInt6sh multiparous females (row 2 vs row 5).

^e^Histologically, a papillary adenocarcinoma

^f^Total mice included 21 heterozygous and 5 homozygous WapInt6sh transgenic mice from J1 founder line that survived 24 months or longer.

^g^p = 0.0016 from the Fisher's exact test of WapInt6sh virgins vs WapInt6sh multiparous females (row 3 vs row 5).

^h^Includes multiparous FVB/N wild-type littermates resulting from WapInt6sh heterozygous crosses. Each mouse is identified in Additional file 3.

^i^p = 0.035 from the Fisher's exact test of wild-type multiparous vs WapInt6sh multiparous females (row 4 vs row 5).

^j^Of the 11 tumors arising in 10 multiparous transgenic females, there were 4 undifferentiated tumors, 5 papillary adenocarcinomas and 2 glandular adenocarcinomas.

^k^Total mice included both heterozygous or homozygous WapInt6sh transgenic mice from J1 founder line that lived to tumor age (>12 months) and produced >1 litter.

**Figure 3:**
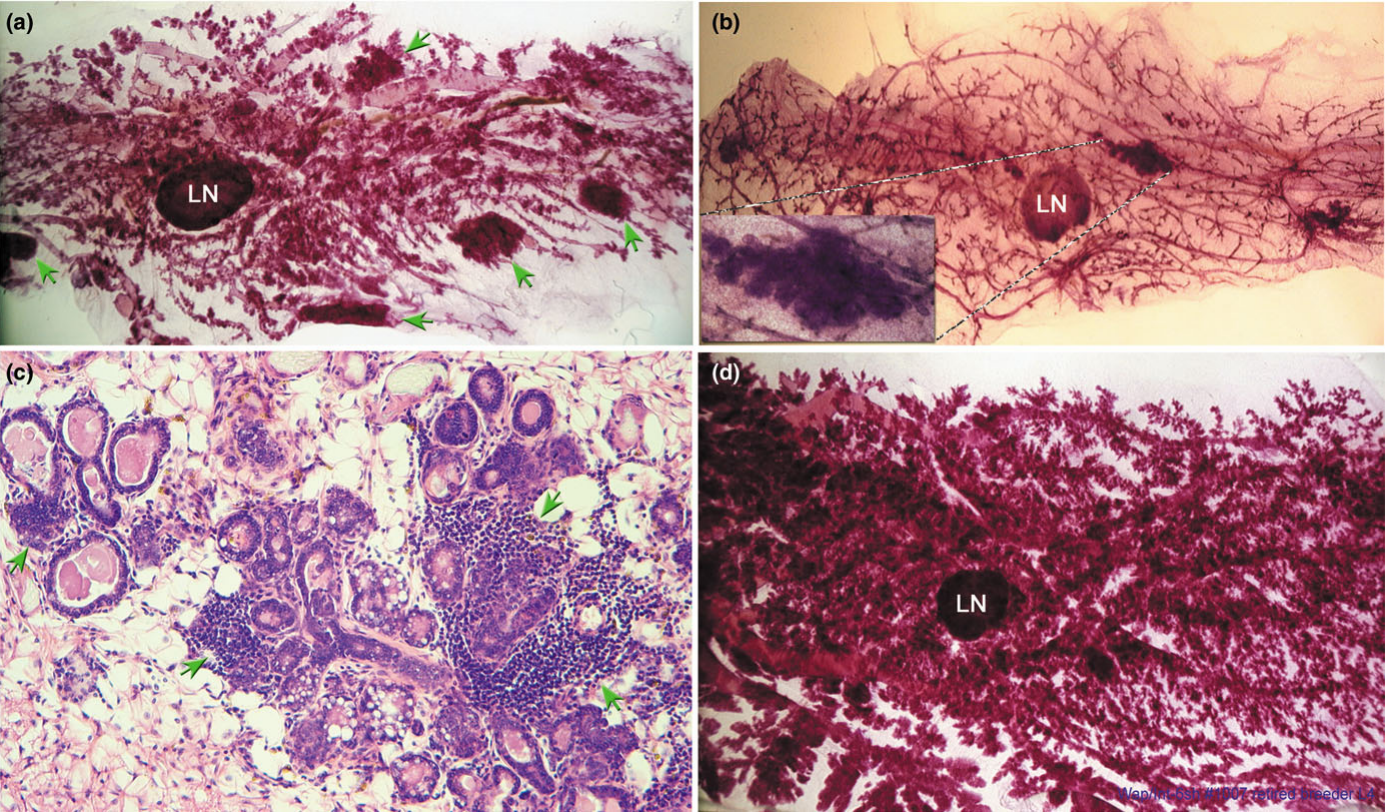
Mammary glands from multiparous WapInt6sh transgenic females show persistent alveolar hyperplasia. **(a) **Multiple focal alveolar hyperplasias (green arrows) are visible in this carmine alum stained whole mounted mammary gland from a multiparous WapInt6sh transgenic female. LN, lymph node. Magnification 10 ×. **(b) **A no. 4 mammary gland from a WapInt6sh multiparous female containing one focal hyperplasia at 10 × magnification. Inset panel shows the hyperplasia at higher magnification (25 ×). This gland and the gland shown in Panel A were each taken from mice with tumors in their contralateral no. 4 mammary glands. **(c) **Hematoxylin and eosin-stained 5 μm section through the focal hyperplasia shown in Panel B. Well defined acinar structures are visible at 100 × magnification surrounded by significant lymphocytic infiltration (green arrows). **(d) **Representative multiparous WapInt6sh mammary gland showing a more uniform persistence of alveolar hyperplasia. This female had three litters, the last of which was weaned 9 months before this gland was harvested.

Multiple focal alveolar hyperplasias were also frequently observed in the non-tumor-containing mammary glands of the same 15–22-month-old retired breeders (Figure 3a,b). The presence of these hyperplastic alveolar nodules (HANs) recapitulated the phenotype observed in the mammary glands of mice harboring the original MMTV insertion in Int6 [1]. Int6sh-induced hyperplasia persisted in multiparous females well after involution (see Figure 3a,b, with 25 × inset, and 25 × magnification of an HAN shown in c) and could be premalignant populations. Higher magnification of HANs frequently showed extensive lymphocytic infiltration (Figure 3c). Approximately 80% of retired Int6sh breeders (with two or more litters) displayed the focal hyperplastic phenotype in an otherwise normally involuted gland (Figure 3a,b). In contrast, mammary glands from the other 20% of Int6sh multiparous females failed to involute at all or showed a marked delay in involution over the whole gland as long as 15 months after their last litter was weaned (Figure 3d). All tumors, regardless of tumor type, arose in mice that had focal or global hyperplasia in their contralateral glands. Additional file 3 documents the frequency and type of hyperplasias in all 24 transgenic mice analyzed.

To more directly demonstrate that the population of cells forming hyperplasia arose from alveolar epithelium surviving involution after lactation, the WapInt6sh mouse was crossed with the WapCreRosa26stop mouse. Previous work from our laboratory identified a LacZ-tagged population of parity-induced mammary epithelial cells (PI-MECs) that is pluripotent and has the ability to self-renew [14]. Committed alveolar cells will also express β-galactosidase after conditional activation by the Cre-lox recombinase, which is driven by the Wap promoter. If both PI-MECs and fully committed alveolar cells survive involution because of Int6sh expression then the number and persistence of LacZ-positive cells following the cessation of lactation should increase. Parous WapCreRosa26stopWapInt6sh females developed foci of LacZ-positive alveolar cells, which survived post-lactation involution confirming that mammary epithelial cells formed during pregnancy persistently survived in WapInt6sh-expressing mammary glands. Additional file 4 shows the no. 3 mammary glands from two different multiparous WapCreRosa26WapInt6sh mice, one at 4 months (a and c) and the other at 6 months of involution (b and d). The X-Gal stained whole mounts (a and b) clearly show multiple LacZ+ focal hyperplasias after several months of involution, similar to the WapInt6sh transgenic. Higher magnification images (c and d, 200 ×) of two different focal areas of hyperplasia show LacZ+ luminal epithelial cells interspersed with LacZ negative cells. In an earlier report, our laboratory demonstrated that PI-MECs were targets for MMTV-ErbB2 induced mammary cancer [19]. Therefore the persistent survival of an alveolar population from one pregnancy through the next represents a premalignant population that exhibits an increased predisposition for tumor development as demonstrated earlier for mice transgenic for mammary-specific transforming growth factor alpha (TGF-α) [16]. In agreement with this hypothesis, WapInt6sh-induced tumor development is accentuated by multiple pregnancies.

### Transgenic Int6sh is expressed in mammary tumors and hyperplasias

**Figure 4:**
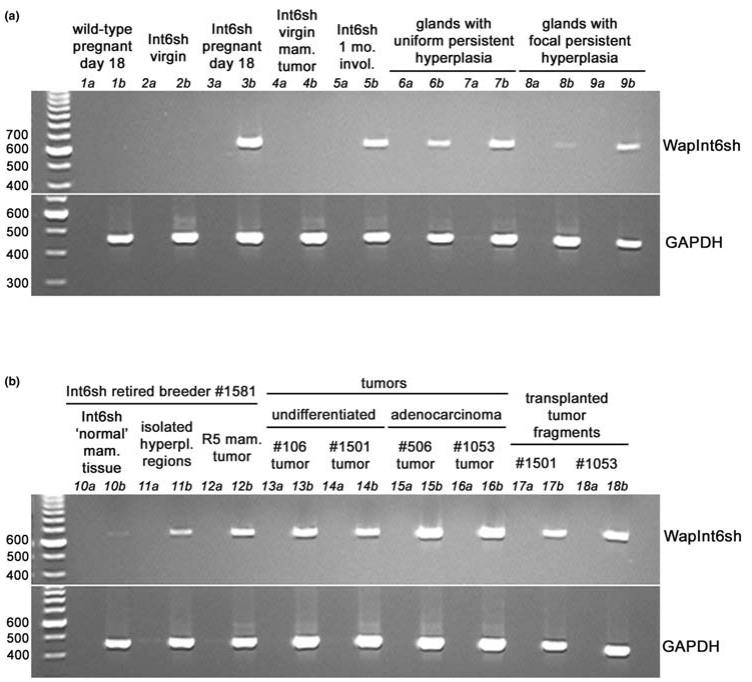
WapInt6sh relative expression levels determined by linear RT-PCR. The top panel shows the relative expression of Int6sh in various wild-type and transgenic mammary glands at different stages of development. Expression of the housekeeping gene, GAPDH is shown for comparison. The level of expression of Int6sh in lanes 6–9 is proportional to the extent of hyperplasia observed in those glands. The bottom panel shows Int6sh expression from dissected portions of the mammary glands of one mouse (no. 1,581, lanes 10–12), from independently arising tumors (lanes 13–16) and from outgrowths arising after transplanting tumor fragments (lanes 17,18). Histologically 'normal' mammary tissue (shown in lanes 10, a and b) was dissected away from surrounding focal hyperplasias and tumor tissue and then pooled. Transplantation of parous WapInt6sh tumor fragments into mammary epithelium-divested fat pads produced outgrowths that expressed detectable amounts of Int6sh RNA, suggesting that they also arose from mammary cells constitutively expressing the transgene (lanes 17,18). First strand reactions without RT and those with RT are designated as a and b respectively.

To determine the relative expression levels of Int6sh mRNA in transgenic mammary glands and tumors semi-quantitative RT-PCR was used. Figure 4a shows the linear amplification of a fragment spanning the Wap promoter and Int6sh transgene junction (see also Figure 1) relative to the expression of the GAPDH housekeeping gene. As expected, Int6sh expression is not present in the wild type (lane 1) nor the virgin mammary glands (lane 2) but is induced in late pregnancy (lane 3b). The tumor arising in a virgin Int6sh transgenic did not express detectable levels of Int6sh (lane 4), suggesting that growth of this tumor is not dependent on Int6sh expression. Uniformly hyperplastic glands and glands containing multiple focal hyperplasias also express varying amounts of Int6sh mRNA (lanes 6–9) indicating that either expression from the Wap promoter is maintained in the lobular structures.

Figure 4b shows the results of Int6sh mRNA amplification from the two non-tumor-containing inguinal mammary glands and a mammary tumor arising in the right no. 5 mammary gland dissected from an Int6sh retired breeder. The surrounding normal tissue showed barely detectable levels of Int6sh while the focal hyperplasias and tumor showed higher expression (lanes 10–12). Likewise, all the tumors, whether undifferentiated or adenocarcinomas, showed high levels of Int6sh expression (lanes 13–16) indicating that the Wap promoter remained constitutively activated both in the tumors and in the alveolar hyperplasia. A high level of Int6sh expression was also observed in hyperplastic outgrowths that resulted after transgenic tumor fragments were transplanted into the epithelium-divested fat pads of non-pregnant, wild-type recipients (lanes 17,18).

### Gene expression profiling of Int6sh-induced hyperplasias and tumors

**Figure 5:**
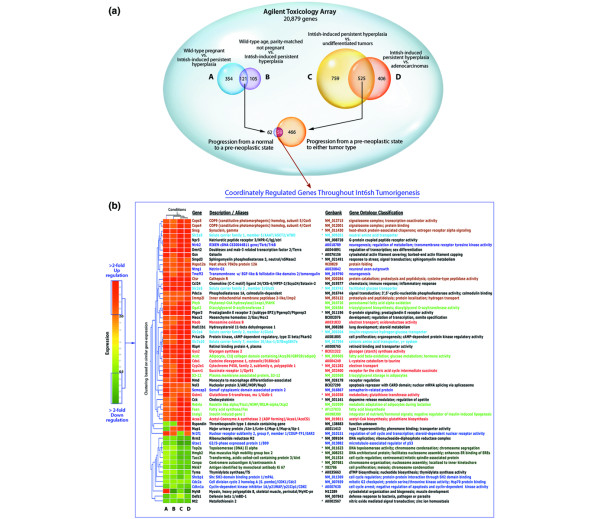
Gene expression profiling of Int6sh-expressing hyperplasias and tumors. **(a) **Total RNA from wild-type, hyperplasia-containing and tumor-containing transgenic mammary glands was prepared and pools were made consisting of five mice each. The following comparisons were performed on Agilent Mouse 22 K 60-mer Oligo Arrays (G4121A): wild-type pregnant mammary glands vs Int6sh transgenic mammary glands at 18 months of age containing persistent hyperplasia; non-pregnant wild-type age/parity-matched mammary glands vs Int6sh persistent hyperplastic glands; Int6sh hyperplastic glands vs undifferentiated tumors and Int6sh hyperplastic glands vs adenocarcinomas. The colored spheres show the number of genes that are at least twofold up or downregulated in each one of these comparisons. The numbers in the intersected spaces denotes genes that are common to any particular pairwise comparison. **(b) **The expression heatmap and gene list at the bottom of the figure shows those genes found to be coordinately regulated throughout Int6sh-induced mammary tumorigenesis. The GeneSpring expression clustering algorithm was employed to determine which genes show the same pattern of expression in the four conditions, denoted A, B, C and D, corresponding to the comparisons shown in **(a)**. The color bar on the left shows how hue relates to direction and extent of the transcriptional differences. Functional groups font color key: dark orange, protein folding and degradation; light blue, solute transporters; purple, neuronal guidance; red, energy metabolism; lime green, adipose, lipid and fatty acid metabolism; dark blue, cell cycle regulation; green, chromatin architecture and chromosome segregation. Asterisks denote genes that have been previously implicated in mammary tumorigenesis.

To establish a potential mechanism for the action of Int6sh in the tumorigenic process, microarray analysis was performed in an attempt to gain insight into what processes or pathways might be altered by the presence of truncated Int6. To accomplish this, mammary RNA from Int6sh-expressing persistent hyperplasia was compared with mammary RNA from age- and parity-matched pregnant and non-pregnant wild-type females in order to detect presumptive gene expression alterations associated with persistent Int6sh-induced hyperplasia. In addition, RNA was extracted from first pregnancy wild-type and Int6sh transgenic mice to determine if the same pattern of gene expression could be detected even earlier, before phenotypic changes were evident. To detect gene expression differences as pre-neoplastic hyperplasia progresses to tumor, the same RNA from Int6sh-expressing hyperplasia was hybridized against RNA from the two Int6sh tumor types. Additional file 5 summarizes the microarray comparisons and sample-pooling scheme. Genes coordinately regulated throughout tumorigenesis were identified by comparing *in silico *the 121-member gene list identified during the pre-neoplastic phase to the 525-member gene list found during the tumor phase. Figure 5a outlines how each comparison was performed and the number of genes identified as at least twofold up and downregulated in each pairwise comparison. Unsupervised hierarchical cluster analysis produced a list of 59 genes that are coordinately regulated during Int6sh-induced tumorigenesis is shown in Figure 5b. Approximately twice as many genes were at least twofold upregulated as downregulated, which might not be surprising given the increased metabolic activity of the tumors. Of the genes identified, 22 (37%) have been previously associated with mammary tumorigenesis (denoted by the asterisks). Using the gene ontology database within the GeneSpring software and gene ontology annotations [20], 38 of the 59 genes could be assigned to seven functional groups with 4–7 members per group. Genes involved in protein degradation, solute transport, neurogenesis, energy metabolism and lipid, adipose, fatty acid metabolism always cluster together as being upregulated while genes involved with cell cycle regulation and chromosome management were consistently downregulated during the progression to pre-neoplastic lesion and then to frank tumor. Interestingly, protein degradation and chromosome management overlap with functions previously associated with full length Int6 in other model organisms. The expression pattern of the 18 transcripts involved with protein turnover, cell cycle regulation and chromosome segregation have been confirmed by quantitative RT-PCR (see Additional file 1 for the results of the qRT-PCR analysis compared to the annotated microarray heat map shown in Figure 5).

The striking correspondence of gene expression patterns from RNA isolated from the initial pregnancy in WapInt6sh glands with those found in RNA from the persistent alveolar hyperplasia and from the tumor sets argues that the hyperplasia is formed from an alveolar epithelial population that survives post-lactational involution and tissue remodeling. Furthermore, this signature pattern was maintained and strengthened in the tumor RNA suggesting strongly that the tumors arise from the hyperplastic alveolar cells.

To validate the microarray data, quantitative RT-PCR was performed on 18 of the 59 Int6sh tumorigenesis signature genes. This subset of genes was chosen because of their role in tumorigenesis in other cell culture-based and transgenic tumor models and because of their association with previously documented Int6 functions. The results of the qRT-PCR are presented in Additional file 1 along with a comparison to the microarray raw numbers that produced the heat map in Figure 5b. Two different endogenous controls (GAPDH and ACTB) were employed to reduce variability that might exist between normal, hyperplastic and tumor-containing mammary tissues. The mRNA quantitation of 14 of these 18 genes closely mirrored the results of the microarray analysis in terms of up or downregulation and fold-change value, while the expression of the remaining four genes were corroborated in direction of change only. In general, the microarray data under estimated the extent of up or downregulation compared to the qRT-PCR for approximately 50% of the genes analyzed. As these genes were chosen for their role in tumorigenesis and their connection to Int6 function, independent of their up or downregulation or fold-change value, it stands to reason that the same degree of validation could then be applied to the remaining 41 genes in the Int6sh signature transcriptome.

## Discussion

Our observations conclusively demonstrate that targeted expression of the Int6sh mutation to mammary epithelium *in vivo *results in a significant increase in mammary cancer risk. These data provide a strong validation of Int6sh as an oncogenic mutation in mammary epithelium. Although large C-terminal deletions often lead to loss of function, the most frequently suggested mechanism for Int6sh action is as a dominantly acting mutation. This idea is consistent with the observation that interruption (and truncation) of the Int6 gene in MMTV-induced hyperplasia and tumors occurs by proviral DNA insertion in several different introns, any of which could produce a dominant allele. Despite this evidence, it remains formally possible that Int6 haploinsufficiency caused by MMTV integration in the originally isolated hyperplastic outgrowth cell line and the two independent tumors was the cause of the premalignant and malignant phenotypes. Frequent loss of heterozygosity (LOH) of Int6 in both breast and non-small cell lung carcinomas (NSCLC) [21,22] and decreased expression of Int6 through hypermethylation of the promoter and first exon in NSCLC [23] suggest that normal Int6 function might be sensitive to the level of expression. However, the transgenic model reported here demonstrates that the targeted expression of a truncated form of Int6 is sufficient to produce persistent mammary hyperplasia and an increased incidence of mammary tumors in a background of normal wild-type Int6 expression. Indeed throughout the Int6sh tumor progression, endogenous Int6 expression is unchanged as shown in its microarray single-gene expression tracing (Additional file 6). Therefore, in our WapInt6sh model of mammary tumorigenesis, haploinsufficiency does not play a role.

The presence of Int6sh RNA has an immediate impact upon the transcriptome of late pregnant mammary tissue as demonstrated by our microarray analysis. Strikingly, the changes manifest in the WapInt6sh mammary RNA at first pregnancy are emboldened in the RNA isolated from the alveolar hyperplasia that persists in WapInt6sh parous females. This indicates that there is preferential survival of Int6sh-expressing alveolar cells following the cessation of lactation. Int6sh-induced tumors arise as a consequence of a persistent alveolar hyperplasia that morphologically resembles pregnant lobular/alveolar mammary tissue. The persistence of this cell type might be linked to a decrease in the rate of apoptosis during involution, consistent with the observation of Mayeur *et al*. that fourfold fewer Int6sh-expressing fibroblasts go through apoptosis compared to wild-type control cells or cells expressing only full-length Int6 [12]. Subsequently, mammary tumors arise in these glands and analysis bears out that the tumor transcriptome bears many features of the normal and hyperplastic Int6sh-expressing tissues. These results provide strong evidence for the linear progression of Int6sh-expressing epithelium from normal through hyperplasia to tumor formation.

The long tumor latency coupled with the observation that the tumors arose stochastically (usually only one gland per mouse was affected) suggests that other genetic or epigenetic events were required for initiation or progression of tumor growth. We therefore compared gene expression in mammary tissues where Int6sh was expressed with expression profiles in age and developmental stage-matched wild-type mammary tissue. The 59 genes comprising the Int6sh-induced tumorigenic gene expression signature include 22 genes that have been previously associated with mammary tumorigenesis (Figure 5).

The Int6sh-induced tumorigenesis gene list contains several genes that participate in cellular processes that have been linked to full length Int6 function in other model systems (Figure 5b). The most notable example is the consistent upregulation of two components of the COP9 signalosome, namely Cops5 (CSN5/Jun activation domain-binding protein 1, Jab1) and Cops4 (CSN4), in both pre-neoplastic mammary glands and tumors. In addition, four other proteins involved with protein turnover (γ-synuclein, 70 kDa heat shock protein 12A, cathepsin R and inner mitochondrial membrane peptidase 2-like) were also consistently upregulated during this tumor progression. Neither CSN5 nor CSN4 have been shown to directly interact with Int6 in the signalosome, but several lines of evidence make their association with Int6sh tumorigenesis intriguing. CSN4 is less well studied but is believed to mediate assembly of the CSN holocomplex through its PCI domain, which it shares with Int6. CSN5 is by far the most studied component of the COP9 signalosome having been linked to tumor initiation and progression through maintenance of DNA fidelity, cell cycle control, DNA repair, regulation of apoptosis, angiogenesis and microenvironmental homeostasis [24].

Several other gene ontological groups showed consistent upregulation throughout Int6sh tumorigenesis, including four closely related solute carrier family members (Slc1a5, Slc2a4, Slc2a5 and Slc7a10) and four neurogenesis/neuronal axon outgrowth molecules (Netrin-G1, Tomoregulin, Semaphorin cytoplasmic domain associated protein 2 and Ntrk2). Ducts within Int6sh-expressing mammary glands, that did not develop tumors, showed multiple morphological defects including decreased secondary branching and termination at blood vessels (data not shown). It is possible that the abnormally high expression of several neuronal guidance transcripts contributes to the defects in allometric ductal growth.

Two consistently downregulated gene ontological groups were also identified during the Int6sh-induced progression from pre-neoplastic lesion to frank tumor. There was a group of five genes involved in cell cycle regulation, with four of the genes always downregulated, including Gtse1 (a microtubule-associated regulator of p53), Shcbp1 (a Shc SH-2 domain binding protein), Cdc2a (also called CDK1, an Hsp70 binding protein involved in the mitotic G2 checkpoint) and Cdkn1a (a cyclin-dependent kinase inhibitor, also called p21/Waf/Cip1). Several studies have linked the CSN to the regulation of the cellular proliferation machinery. Molecules connecting these two processes include the cyclin-dependent kinase inhibitors p27/Kip1 [25] and p21/Waf/Cip1 [26], as well as cyclins D1 [27,28] and E [29,30]. In addition, Kato and colleagues recently showed that in the CSN5 knock-out there were elevated levels of the cell cycle regulatory genes p27/Kip1, p53 and cyclin E [31]. As a result, cell proliferation is impaired and apoptosis is accelerated. However, in our tumorigenic model CSN5 is upregulated as a result of Int6sh expression, possibly causing a decrease in p21 (as observed), p53 and cyclin E levels leading to increased proliferation and survival of a population of mammary epithelial cells.

## Conclusion

The Int6sh transgenic mouse model presented here provides the first and to date only *in vivo *evidence that the expression of truncated Int6 leads to persistent mammary hyperplasia and increased predisposition to mammary tumors. The immediate effect that expression of truncated Int6 has on the transcriptome of the differentiating alveolar mammary epithelium suggests that further study regarding this aspect of Int6sh expression could provide a useful mechanism for understanding the complex function(s) of Int6 in translation initiation, the COP9 signalosome and the proteasome.

## Abbreviations

COP9 = constitutive photomorphogenesis 9 signalosome (CSN); eIF3 = eukaryotic translation initiation factor 3; GAPDH = glyceraldehyde 3-phosphate dehydrogenase; GFP = green fluorescent protein; HAN = hyperplastic alveolar nodule; LOH = loss of heterozygosity; LTR = long terminal repeat; MMTV = Mouse mammary tumor virus; MPN = Mpr-1-Pad-1-N-terminal domains; NLS = nuclear localization sequence; NSCLC = non-small cell lung carcinoma; PCI/PINT = 26S proteasome-COP9 signalosome-initiation factor 3/proteasome subunits-Int6-Nip-1-TRIP-15; RT-PCR = reverse transcriptase-polymerase chain reaction; Wap = whey acidic protein.

## Competing interests

The authors declare that they have no competing interests.

## Authors' contributions

DM carried out phenotypic characterization including histology, Int6sh mRNA expression and microarrray analyses and drafted the manuscript. CB made some of the early phenotypic observations. RC created the transgenic construct and edited the manuscript. GS conceived the study (along with RC), participated in its design and coordination and helped draft the manuscript. All authors read and approved the final manuscript.

## Supplementary Material

Additional file 1 Excel file showing results of qRT-PCR analysis on 18 of the 59 Int6sh tumorigenesis signature genes. Click here for file

Additional file 2 Tiff file showing how the relative expression of Int6sh was assessed during mammary gland development by semi-quantitative RT-PCR. Click here for file

Additional file 3 Word documents giving a summary of Int6sh-induced hyperplasia and tumor incidence. Click here for file

Additional file 4 Tiff file showing **(a) **and **(b)**: X-Gal stained whole mounts of WapCreRosa26stopWapInt6sh involuted no. 3 mammary glands (10 × magnification). **(c) **and **(d)**: higher magnification (200 ×) of 6 μm sections of the mammary glands shown in **(a) **and **(b)**. **(c) **is a photo of one of the hyperplasias shown in panel A with a similar relationship between **(d) **and **(b)**. Several of the larger LacZ+ focal hyperplasias are shown by orange arrows. Click here for file

Additional file 5 Word document giving a detailed summary of the microarray comparisons and mammary gland total RNA sample pooling scheme. Click here for file

Additional file 6 Tiff file showing endogenous Int6 expression tracing generated from a single gene query of the microarray data. The upper left portion shows the various Int6 unique identifiers and gene ontology designations. The table in the upper right shows the raw expression values produced from each pair wise comparison shown in the Venn diagram in Figure 5a. The bottom panel shows the tracing of full length Int6 expression as the premalignant lesions progress to tumors. Click here for file
