# Supplementary figures and images for: Expression of truncated Int6/eIF3e in mammary alveolar epithelium leads to persistent hyperplasia and tumorigenesis
Source: Breast Cancer Res. 2007 Jul 12;9(4):R42. doi: 10.1186/bcr1742 (PMC2206715; doi:10.1186/bcr1742)

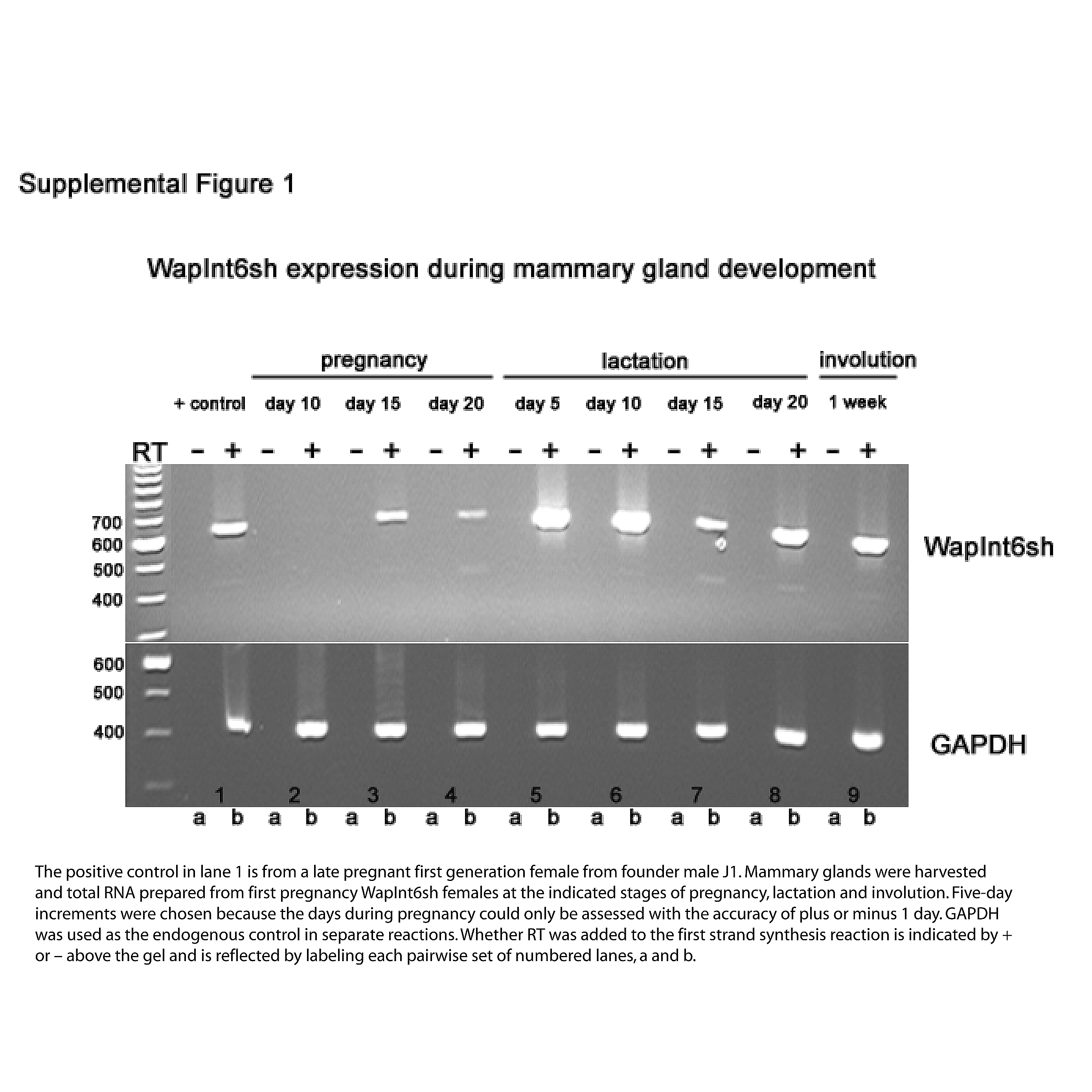

Supplement: Additional file 2 — Tiff file showing how the relative expression of Int6sh was assessed during mammary gland development by semi-quantitative RT-PCR. [file bcr1742-S2.tiff]

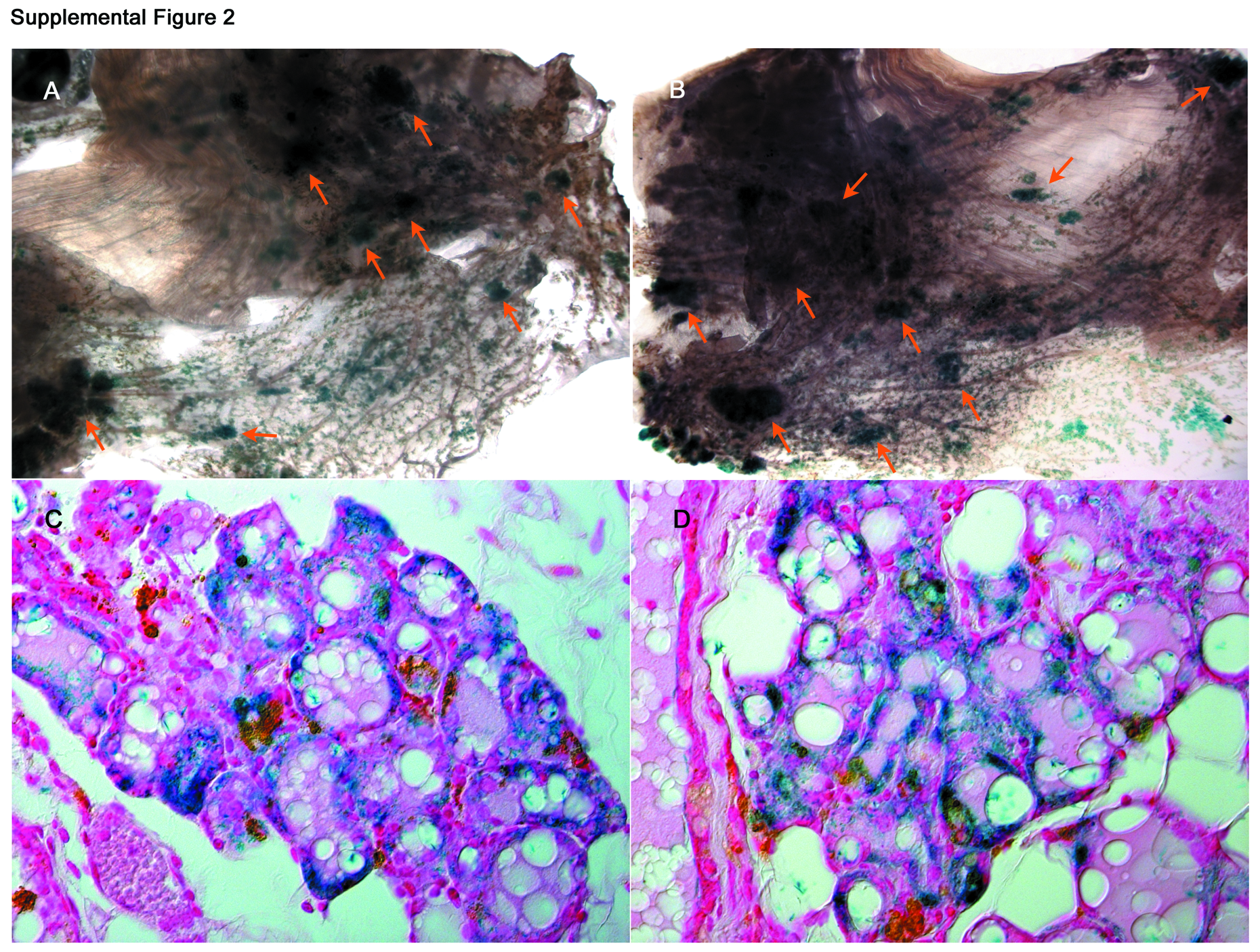

Supplement: Additional file 4 — Tiff file showing (a) and (b): X-Gal stained whole mounts of WapCreRosa26stopWapInt6sh involuted no. 3 mammary glands (10 × magnification). (c) and (d): higher magnification (200 ×) of 6 μm sections of the mammary glands shown in (a) and (b). (c) is a photo of one of the hyperplasias shown in panel A with a similar relationship between (d) and (b). Several of the larger LacZ+ focal hyperplasias are shown by orange arrows. [file bcr1742-S4.tiff]

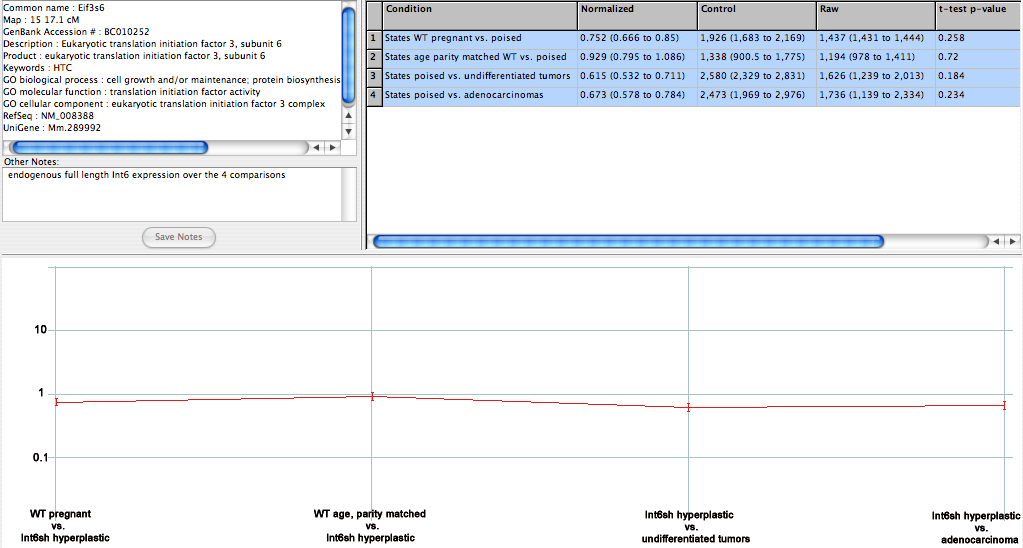

Supplement: Additional file 6 — Tiff file showing endogenous Int6 expression tracing generated from a single gene query of the microarray data. The upper left portion shows the various Int6 unique identifiers and gene ontology designations. The table in the upper right shows the raw expression values produced from each pair wise comparison shown in the Venn diagram in Figure 5a. The bottom panel shows the tracing of full length Int6 expression as the premalignant lesions progress to tumors. [file bcr1742-S6.tiff]
