# Supplementary material for: Expression of truncated Int6/eIF3e in mammary alveolar epithelium leads to persistent hyperplasia and tumorigenesis
Source: Breast Cancer Res. 2007 Jul 12;9(4):R42. doi: 10.1186/bcr1742 (PMC2206715; doi:10.1186/bcr1742)
Supplement: Additional file 3 — Word documents giving a summary of Int6sh-induced hyperplasia and tumor incidence. [file bcr1742-S3.doc]

Supplemental Table 1

Incidence of Int6sh-induced hyperplasia and tumors

| Mouse number^a^ | Genotype | Hyperplasia^b^ | Mammary  Tumor^c^ | Tumor  Location^d^ | Age at  Analysis  (months)^e^ | Number of  Litters | Microarray  Samples^f^ |
| --- | --- | --- | --- | --- | --- | --- | --- |
| 106 | WapInt6sh+ | focal | U | R3 | 16 | 3 | Pool 4 |
| 506 | WapInt6sh+ | focal | PA | L4 | 14 | 3 | Pool 5 |
| 1006 | WapInt6sh+ | focal | PA | L4 | 19 | 2 | Pool 5 |
| 1007 | WapInt6sh+ | mixed | -- | -- | 20 | 2 |  |
| 1039 | WapInt6sh+ | focal | -- | -- | 18 | 2 |  |
| 1053 | WapInt6sh+ | focal | PA | L4 | 18 | 4 | Pool 5 |
| 1053 | WapInt6sh+ | focal | GC | L3 | 18 | 4 |  |
| 1172 | WapInt6sh+ | focal | -- | -- | 17 | 3 | Pool 1 |
| 1186 | WapInt6sh+ | mixed | -- | -- | 17 | 4 | Pool 1 |
| 1196 | WapInt6sh+ | focal | -- | -- | 19 | 2 |  |
| 1197 | WapInt6sh+ | uniform | -- | -- | 20 | 4 |  |
| 1199 | WapInt6sh+ | focal | -- | -- | 20 | 2 |  |
| 1501 | WapInt6sh+ | focal | U | L4 | 18 | 2 | Pool 4 |
| 1549 | WapInt6sh+ | mixed | -- | -- | 11 | 2 |  |
| 1580 | WapInt6sh+ | uniform | U | L4 | 14 | 3 | Pool 4 |
| 1581 | WapInt6sh+ | focal | GC | R5 | 16 | 3 |  |
| 1583 | WapInt6sh+ | uniform | -- | -- | 13 | 2 |  |
| 1668 | WapInt6sh+ | focal | -- | -- | 12 | 4 |  |
| 1672 | WapInt6sh+ | focal | -- | -- | 15 | 3 | Pool 1 |
| 1680 | WapInt6sh+ | focal | -- | -- | 15 | 2 | Pool 1 |
| 1684 | WapInt6sh+ | uniform | -- | -- | 15 | 2 | Pool 1 |
| 9377 | Wild-type | none | -- | -- | 19 | 3 | Pool 3 |
| 9457 | Wild-type | none | -- | -- | 17 | 2 | Pool 3 |
| 9462 | Wild-type | none | -- | -- | 19 | 3 | Pool 3 |
| 9492 | Wild-type | none | -- | -- | 14 | 3 | Pool 3 |
| 9558 | Wild-type | none | -- | -- | 15 | 2 | Pool 2 |
| 9562 | Wild-type | none | -- | -- | 15 | 3 | Pool 2 |
| 9566 | Wild-type | none | -- | -- | 16 | 2 | Pool 2 |
| 9575 | Wild-type | none | -- | -- | 13 | 2 |  |
| 12506 | WapInt6sh+ | focal | PA | R4 | 18 | 4 |  |
| 12507 | WapInt6sh+ | focal | U | R4 | 21 | 5 |  |
| 12508 | WapInt6sh+ | uniform | PA | R4 | 15 | 3 | Pool 5 |
| 12544 | WapInt6sh+ | uniform | -- | -- | 22 | 4 |  |

^a^Note: mouse no. 1,053 appears twice because it had two tumors.

^b^The type of hyperplasia, either focal or uniform, as found in the non-tumor containing no. 3 or no. 4 mammary glands. Mixed denotes the presence of both types of hyperplasia in the same gland. All multiparous Int6sh mice examined contained hyperplastic lesions.

^c^Tumor type: U, undifferentiated; PA, papillary adenocarcinoma; GC, glandular carcinoma.

^d^R3, right no. 3 mammary gland; L3, left no. 3 mammary gland; R4, right no. 4 mammary gland; L4, left no. 4 mammary gland; R5, right no. 5 mammary gland.

^e^Age of each mouse was rounded to the nearest whole month.

^f^Additional file 1 describes how samples were chosen and pools combined for microarray analysis.
