# Supplementary material for: Expression of truncated Int6/eIF3e in mammary alveolar epithelium leads to persistent hyperplasia and tumorigenesis
Source: Breast Cancer Res. 2007 Jul 12;9(4):R42. doi: 10.1186/bcr1742 (PMC2206715; doi:10.1186/bcr1742)
Supplement: Additional file 5 — Word document giving a detailed summary of the microarray comparisons and mammary gland total RNA sample pooling scheme. [file bcr1742-S5.doc]

Supplemental Table 2:

Detailed summary of the microarray comparisons and mammary gland total RNA sample pooling scheme.

| Microarray number | Cy3 | Cy5 |
| --- | --- | --- |
| 1 | L4 and R4 mammary glands from WapInt-6sh persistent hyperplasias ; ~16 months old; multiparous (2-5 litters); at least 6 months since last litter; “poised” to develop tumors by 18 months of age.  **[POOL #1 = mouse #’s 1172, 1186, 1672, 1680, 1684]** | L4 and R4 mammary glands from multiparous wild-type FVB (3-5 litters) currently pregnant; harvested at late pregnancy (day 17-19) or early lactation.  **[POOL #2 = mouse #’s 9562, 9558, 9566]** |
| 2 | Dye swap of microarray #1: Multiparous wild-type FVB (3-5 litters) currently pregnant; harvested at late pregnancy (day 17-19) or early lactation.  **[POOL #2]** | Dye swap of microarray #1: L4 and R4 mammary glands from Wap/Int-6sh persistent hyperplatic pool; ~16 months old; multiparous (2-5 litters); at least 6 months since last litter; “poised” to develop tumors by 18 months of age.  **[POOL #1]** |
| 3 | L4 and R4 mammary glands from WapInt-6sh persistent hyperplasias; ~16 months old; multiparous (2-5 litters); at least 6 months since last litter; “poised” to develop tumors by 18 months of age.  **[POOL #1]** | L4 and R4 mammary glands from age- and parity-matched wild-type control FVB mice; 3-8 months since last litter, fully involuted? .  **[POOL #3 = mouse #’s 9492, 9377, 9462, 9457]** |
| 4 | Dye swap of microarray # 3: L4 and R4 mammary glands from age- and parity-matched wild-type control FVB mice; 3-8 months since last litter, fully involuted? .  **[POOL #3]** | Dye swap of microarray #1: L4 and R4 mammary glands from Wap/Int-6sh persistent hyperplatic pool; ~16 months old; multiparous (2-5 litters); at least 6 months since last litter; “poised” to develop tumors by 18 months of age.  **[POOL #1]** |
| 5 | Dye swap of microarray #1: L4 and R4 mammary glands from Wap/Int-6sh persistent hyperplatic pool; ~16 months old; multiparous (2-5 litters); at least 6 months since last litter; “poised” to develop tumors by 18 months of age.  **[POOL #1]** | ‘Undifferentiated’ tumors  from Wap/Int-6sh retired breeders  **[POOL #4 = mouse #’s 106, 1580, 1501]** |
| 6 | Dye swap of microarray # 5:  ‘Undifferentiated’ tumors  from Wap/Int-6sh retired breeders  **[POOL #4]** | Dye swap of microarray #1: L4 and R4 mammary glands from Wap/Int-6sh persistent hyperplatic pool; ~16 months old; multiparous (2-5 litters); at least 6 months since last litter; “poised” to develop tumors by 18 months of age.  **[POOL #1]** |
| 7 | Dye swap of microarray #1: L4 and R4 mammary glands from Wap/Int-6sh persistent hyperplatic pool; ~16 months old; multiparous (2-5 litters); at least 6 months since last litter; “poised” to develop tumors by 18 months of age.  **[POOL #1]** | Papillary adenocarcinomas  from Wap/Int-6sh retired breeders  **[POOL #5 = mouse #’s 1053 L4, 1006, 12508, 506]** |
| 8 | Dye swap of microarray # 7:  Papillary adenocarcinomas  from Wap/Int-6sh retired breeders  **[POOL #5]** | Dye swap of microarray #1: L4 and R4 mammary glands from Wap/Int-6sh persistent hyperplatic pool; ~16 months old; multiparous (2-5 litters); at least 6 months since last litter; “poised” to develop tumors by 18 months of age.  **[POOL #1]** |
| 9 | FVB wild-type late **first** pregnancy  **[POOL #6 = mouse #’s 9571, 9581, 9586, 9587]** | Wap/Int-6sh transgenic late **first** pregnancy  **[POOL #7 = mouse #’s 1263, 1255, 1256, 1264]** |
| 10 | Dye swap of microarray # 9:  Wap/Int-6sh transgenic late **first** pregnancy  **[POOL #7]** | Dye swap of microarray # 9:  FVB wild-type late **first** pregnancy  **[POOL #6 ]** |
